# Molecular epidemiology and genetic characterization of PCV2 and PCV3 circulating in domestic pigs and wild boars in central-southern regions of Italy

**DOI:** 10.1186/s12917-025-04928-0

**Published:** 2025-07-21

**Authors:** Irene Melegari, Shadia Berjaoui, Barbara Secondini, Valentina Curini, Valeria Di Lollo, Umberto Molini, Alessio Lorusso, Giovanni Franzo

**Affiliations:** 1 Istituto Zooprofilattico Sperimentale dell’Abruzzo e del Molise, Teramo, 64100 Italy; 2 Department of Animal Medicine, Production and Health, University of Padova, Legnaro, 35020 Italy

**Keywords:** Circovirus, Pig, Wild boar, Lymph node, Italy, NGS

## Abstract

**Background:**

Porcine circoviruses (PCVs) are significant pathogens impacting swine health globally. In Italy, both viruses have been reported to circulate significantly in commercial and rural farms as well as in wild boar populations. However, most information to date originates from studies conducted in Northern Italy, where the majority of pig production is concentrated, while limited data are available from Central Italian regions. This study aimed to address the knowledge gap regarding PCV epidemiology in Central Southern Italy, an area characterized, mainly, by small-scale family, traditional pig farming systems.

**Results:**

Between March 2020 and March 2024, 362 samples were collected and tested using real-time PCR. Positive samples were further characterized by next generation sequencing (NGS) and phylogenetic analysis. The 47.1% ( 95% CI 95% CI: 40.9-53.38%) of the samples were positive for PCV2, with a higher prevalence in wild boars (62.07%; 95% CI 95% CI: 52.19–70.91%) compared to domestic pigs (34.97%; 95% CI: 27.19–43.38%). For PCV3, 38% ( 95% CI: 28.48–48.25%), of the tested animals were positive, with wild boars again showing a higher prevalence (45.16%; 95% CI: 32.48–58.32%).) compared to domestic pigs (26.32%; 95% CI: 13.40–43.10%). Phylogenetic analysis revealed significant genetic variability and confirmed the dominance of the PCV2d genotype. Comparison with other Italian strains highlighted extensive regional circulation and connections between wild and domestic populations. Sequences of this study frequently clustered with strains from the densely pig-populated areas of Northern Italy, supporting multiple long-range introduction events likely mediated by domestic pig trade.

**Conclusions:**

The study underscores the importance of continuous monitoring of PCVs to manage their impact on developing pig farming systems and mitigate potential biosecurity risks associated with interactions between wild and domestic animal populations.

**Supplementary Information:**

The online version contains supplementary material available at 10.1186/s12917-025-04928-0.

## Background

Porcine Circoviruses, particularly PCV2 and PCV3, are significant pathogens within the *Circoviridae* family, genus *Circovirus*, affecting swine populations globally. Circoviruses are small, non-enveloped viruses with circular single-stranded DNA genomes [1]. PCV2 has been especially impactful due to its association with various diseases in pigs, collectively termed porcine circovirus diseases (PCVD), which include PCV2-systemic disease (PCV2-SD, formerly known as postweaning multisystemic wasting syndrome, PMWS), reproductive disease (PCV-RD) and porcine dermatitis and nephropathy syndrome (PDNS) [2]. While the subclinical infection (PCV-SI) has always been the dominating outcome, it was not noticed until vaccines reached the market. The relevance of these forms cannot be neglected since, when not controlled, their impact has been estimated to be overall higher the clinical outcomes [2].

PCV3, a more recent discovery in 2015 [3], is still under investigation and its pathogenicity has long been debated. More recently, it has been consistently linked to reproductive disease (PCV3-RD) as well as multisystemic inflammation in pigs (PCV3-SD) [4].

The genomic organization of both PCV2 and PCV3 is similar to other circoviruses, containing two main open reading frames (ORFs): ORF1, which encodes the replication-associated protein (Rep), crucial for viral replication, and ORF2, which encodes the capsid protein (Cap) and is the major structural protein involved viral attachment and in immune recognition. PCV2 and PCV3 also have other non-structural proteins that contribute to their regulation of their cycle, apoptosis, interaction with the host cell and immune response and definitively, virulence [5].

Despite their shared genomic structure, PCV2 and PCV3 show significant genetic divergence, with PCV3 demonstrating only about 40% sequence similarity to PCV2.

In both viruses, ORF2 is featured by the higher genetic variability and in thus the most sequenced region and target for strain classification and molecular epidemiology studies.

PCV2 is formally divided into 8 genotypes, with the most common being PCV2a, PCV2b, and PCV2d [6, 7]. Historically, PCV2a and thereafter PCV2b (first genotype shift) were predominant, but in recent years, PCV2d has emerged as the most widespread genotype in many regions (second genotype shift), although more recent evidences demonstrated much more complex spatio-temporal patters [7]. All genotypes have been detected in multiple countries, with major genotypes demonstrating a worldwide distribution, persisting over decades and forming a complex network of connections at both national and continental levels. The presence of the same strains and genotypes across different countries, along with the coexistence of multiple strains and genotypes within individual countries, suggests that current control measures are largely ineffective [7]. PCV3, on the other hand, has shown less genomic diversity and only one genotype is currently recognized, though a certain within-genotype variability has been proven [8]. Also in this case, extensive viral circulation at the global level was demonstrated, with strains collected from different countries showing close genetic and phylogenetic relationships [8].

In Italy, as in many other swine-producing countries, PCV2 has been widely reported, with the PCV2d genotype being the most prevalent in recent years [9]. Vaccination programs have been effective in reducing the clinical impact of PCV2; however, the virus remains endemic, and subclinical infections are common [10, 11]. Surveillance of PCV3 is less implemented, but studies suggest its presence in Italian swine herds, prompting further research to understand its epidemiology and potential impact on pig health in the country [12]. For both viruses a significant circulation in wild boar and rural farms has been reported and these niches seem to have a significant role, although different, in both virus epidemiology [9, 12–14].

However, at the present state, most information originates from studies performed in Northern Italy, where most of the pig production is located, while few data are available from Central-Southern Italy regions [15]. Although less economically relevant compared to the Northern pig farming, Central-Southern production is developing and featured by some peculiarities that characterize it, but also exposed to potential epidemiological threats. In Central-Southern Italy, pig farming is largely small-scale family consisting of no more than four animals, intended for self-consumption and the production of specific regional foods. Only a smaller part is composed of small and large-scale commercial indoor intensive farms for fattening purposes [16].,. Many small-scale family farms adopt semi-free ranging systems, allowing animals to live outdoors most of the year, which promotes animal welfare and sustainable practices. However, outdoor farming also poses a significant risk of disease transmission, which is a key concern highlighted in epidemiological surveys on small and medium-sized farms [17]. The industry is closely tied to traditional food products, with several products awarded Protected Denomination of Origin (PDO) and Protected Geographical Indication (PGI) status, highlighting their cultural significance.

The purpose of this study was to contribute to fill this gap, investigating the frequency of PCVs in domestic and wild pig populations and characterizing them, to unravel the epidemiological connections with other Italian regions.

## Materials and methods

### PCV2 and PCV3 detection

Archive samples originating from the diagnostic activity performed by the Istituto Zooprofilattico Sperimentale dell’Abruzzo e del Molise between March 2020 and March 2024 were included in the studies. Three hundred and sixty two samples, consisting of heart, intestine, lung, spleen, brain, lymph nodes, kidney, placenta and serum collected from 259 animals, 143 domestic pigs and 116 wild boars, located in 3 different regions of Central Italy (Abruzzo, Molise and Marche) (Table 1) had been sampled and delivered from farm veterinarians and public authorities for diagnostic purposes or surveillance activities, respectively. Age and clinical history data were available only for 10% of the swine tested, which were all post-weaning pigs and showed severe respiratory symptoms or died unexpectedly. No clinical history was available for wild boars, as animals of all ages were found dead hit by cars; Most of the sampled farms were small-scale family operations, with the majority of tested animals being in the fattening stage. While all samples were tested for PCV2, only those collected between 2023 and 2024, as well as samples collected prior to 2023 that had previously tested positive for PCV2 and were subsequently stored in the institution’s biobank, were tested for PCV3 (Supplementary Table 1). All tissue samples were homogenized using a TissueLyser LT (Qiagen, Germany). An aliquot of homogenized sample was diluted 1:10 in Phosphate-Buffered Saline (PBS) with antibiotics and centrifuged at 1250 g for 15 min at + 4 °C. Total genomic DNA was extracted from the supernatant of the homogenized samples using MagMAX™ CORE Nucleic Acid Purification Kit (Applied Biosystems™, ThermoFischer Scientific, Waltham, MA, USA), following the manufacturer’s instructions and kept at − 80 °C until testing. To assess the presence of PCV2 and PCV3 DNA, a Real Time PCR targeting two conserved fragments of the cap gene [187 nucleotides (nt) and 158 nt respectively] was performed as previously described [18].

### Next generation sequencing and genome assembly

The complete genome of PCV2 positive samples was amplified using four different PCR assays based on the following primer pairs: P1 (forward) 5’-TAATCCTTCCGAAGACGAGC-3’ and P1 (reverse) 3’-CGATCACACAGTCTCAGTAG-5’, P2 (forward) 5’- CAGAAGCGTGATTGGAAGAC-3’ and P2 (reverse) 3’- ATGTAGACCACGTAGGCCTC-5’, P3 (forward) 5’- AGAAGCTCTTTATCGGAGGA-3’ and P3 (reverse) 3’- AAGCGAACCACAGTCAGAAC-5’ P4 (forward) 5’- CTAGAATAACAGCACTGGAG-3’ and P4 (reverse) 3’- GTTCGTCCTTCCTCATTACC-5’ in order to obtain four overlapping fragments [19]. All PCRs were set up with Q5^®^ High Fidelity DNA Polymerase (New England Biolabs, Ipswich, MA, USA) in 50µl reaction: 10 µl of 5X Q5 Reaction Buffer, 1 µl of dNTPs mix [10mM], 2 µl of each primer [10 nm], 0.5 µl of Q5 High Fidelity DNA polymerase [2UI/µl], 10 µl of 5X q5 Hugh Enhancer, 7.5 µl nuclease free water and 5 µl nucleic acid extract. Amplification reactions were performed with the following conditions: one cycle of 98°C for 1 min and 40 cycles of 98°C for 10 s, 55°C for 30s and 72°C for 90s and a final extension of 72°C for 2 min. PCV3 positive samples underwent three different PCRs with the following primer pairs: PCV3_74_F (forward) 5’- CACCGTGTGAGTGGATATAC − 3’ and PCV3_927_R (reverse) 3’- CAAACCCACCCTTAACAG − 5’ (pair 1); PCV3_1303_F (forward) 5’- ACCGGAGGGGTCAGATTTA − 3’ and PCV3_541_R (reverse) 3’- GAGCTGCTGCTTGAAGATCC − 5’ (pair 2), PCV3_817_F (forward) 5’- GTTATAATGGGGAGGGTGCT − 3’ and PCV3_1647_R (reverse) 3’- GCCTGGACCACAAACAC − 5’ (pair 3) to obtain three overlapping fragments to generate the full-length sequence of the PCV3 genome [20]. Briefly, the 50 µl PCR reaction mixture for each reaction contained 10 µl of 5X Q5 Reaction Buffer, 1 µl of dNTPs mix [10 mM], 3 µl of primer [10 nm], 0.5 µl of Q5 High Fidelity DNA polymerase, 10 µl of 5X Q5 High Enhancer, 5.5 µl of nuclease free water and 5 µl of nucleic acid extract. PCR conditions were: 30 s at 98 °C and 45 cycles of 98 °C for 10s, 60 °C for 30s, 72 °C for 90s with a final extension of 72 °C for 5 min. PCR products were purified using a QIAquick PCR Purification Kit (Qiagen, Hilden, Germany) and amplicons of the same sample were mixed and quantified using the Qubit^®^ DNA HS Assay Kit (Thermo Fisher Scientific, Waltham, MA, USA). Samples with at least 3.5 ng/µl of DNA were processed for Whole Genome Sequencing (WGS). Libraries were prepared using Illumina DNA Prep kit (Illumina Inc., San Diego, CA) following the manufacturer’s instructions. Deep sequencing was performed with the NextSeq 2000 platform (Illumina Inc., San Diego, CA, USA) using the NextSeq 1000/2000 P1 300-cycle reagent cartridge 30Gb and P1 FlowCell NextSeq 1000/2000 30 Gb and standard 150 bp paired-end reads (Illumina Inc., San Diego, CA, USA). Bioinformatic analysis was carried out at the Italian National Reference Centre for WGS of microbial pathogens (GENPAT) at IZSAM. After quality check and trimming of raw reads data using FastQC v0.11.5 and Trimmomatic v0.36 [21], respectively, host depletion was performed by Bowtie2 mapper [22]. Fastq files generated were *de novo* assembled using SPADES v3.11.1 [23] (Algorithmic Biology Lab, St Petersburg, Russia), and the contigs obtained were analyzed with BLASTn to identify the best match reference. Mapping of the trimmed reads was then performed using the iVar v1.3 [24]. computational tool to obtain the consensus sequence.

### Phylogenetic analysis

The obtained PCV2 sequences were aligned to a reference dataset recommended by Franzo and Segalés [7] for genotyping through phylogenetic analysis using IQ-Tree [25]. Given the coding nature of ORF2, alignment was conducted at the amino acid level using the MAFFT method [26], and the nucleotide sequences were subsequently overlaid using TranslatorX [27].

Recombination analysis was conducted with RDP4 [28], initially applying the RDP, GENECONV, MaxChi, and 3Seq methods for a preliminary scan, followed by all implemented methods to refine recombination detection. Each method’s settings were adapted to the dataset characteristics as specified in the RDP4 manual. A recombination event was accepted only if detected by at least two methods with a significance level below 0.001 (Bonferroni-corrected) and was then excluded from the dataset. GARD was used to confirm the absence of residual recombination analysis [29].

Phylogenetic relationships among the PCV2d Italian strains were assessed by aligning the sequences obtained in the present study with that described in Faustini et al. [9] and with sequences submitted in Genbank (accessed 15/09/2024). The phylogenetic analysis was performed in IQ-tree, where the optimal substitution model was selected based on the lowest Akaike Information Criterion. The robustness of inferred clades was evaluated through 10,000 ultrafast bootstrap replicates.

PCV3 sequences were analysed at both ORF2 and complete genome levels using a similar approach, comparing them with other Italian strains benefitting from the Italian sequence dataset available in Franzo et al. [12].

## Results

### Diagnosis of PCV2 and PCV3

**Fig. 1:**
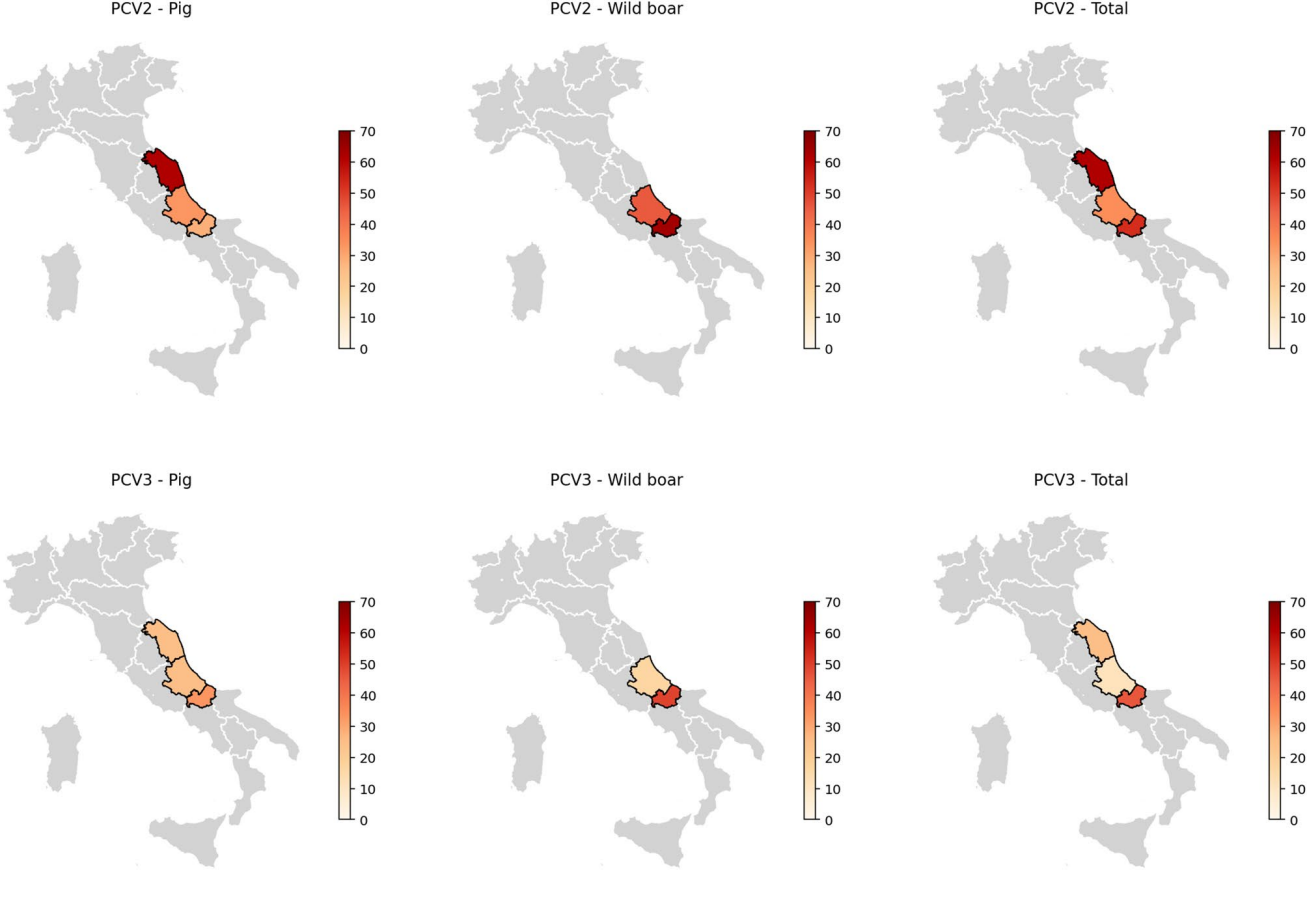
Geographic distribution of PCV2 and PCV3 positivity rates in pigs and wild boars across Abruzzo, Molise and Marche. Each map displays the percentage of PCR-positive samples for either PCV2 (top row) or PCV3 (bottom row), stratified by host species: domestic pigs, wild boars, and the total across both hosts (columns). Data are represented as color gradients, with darker shades indicating higher detection rates

Of the 259 animals tested for PCV2, 122 tested positive (122/259, 47.1%; 95% confidence interval (95% CI): 40.9-53.38%), including 50 domestic pigs (50/143, 34.97%; 95% CI: 27.19–43.38%) and 72 wild boars (72/116, 62.07%; 95% CI: 52.19–70.91%). Among the 100 animals also tested for PCV3, 38 were positive (38/100, 38%; 95% CI: 28.48–48.25%), comprising 10 domestic pigs (10/38, 26.32%; 95% CI: 13.40–43.10%) and 28 wild boars (28/62, 45.16%; 95% CI: 32.48–58.32%). Of these 38, 9 were positive only for PCV3 (9/38, 23.68%; 95% CI: 11.44–40.24%), including 4 domestic pigs (4/38, 10.53%; 95% CI: 2.94–24.91%) and 5 wild boars (5/62, 8.06%; 95% CI: 2.67–17.83%) (Table 2). Additionally, 29 animals were coinfected with both PCV2 and PCV3 (29/100, 29%; 95% CI: 20.36–39.93%), including 6 domestic pigs (6/38, 15.79%; 95% CI: 3.31–18.22%) and 23 wild boars (23/62, 37.1%; 95% CI: 25.16–50.31%) (Table 1). The only organs co-infected by PCV2 and PCV3 were intestine and lungs, with 22 out of 61 intestine (22/61, 36,07%; 95% CI: 24.16–49.37%) and 6 out of 34 lungs tested (6/34, 17.65%; 95% CI: 6.76–34.53%), respectively. For PCV2, in the Abruzzo region, 33 out of 95 sampled subjects tested positive (34.74%; 95% CI: 25.26–45.20). In the Molise region, 80 out of 151 animals tested positive (80/151, 52.98%; 95% CI: 25.26–45.20), while in the Marche region, 8 out of 13 domestic pigs (61.54%; 95% CI: 31.58–86.14) were found positive. PCV3 was found in domestic pigs and wild boards in Abruzzo and Molise regions while in Marche region the virus was detected only in 2 domestic pigs (Table 1; Fig. 1).

### PCV2 full genome sequencing and phylogenetic analysis

**Fig. 2:**
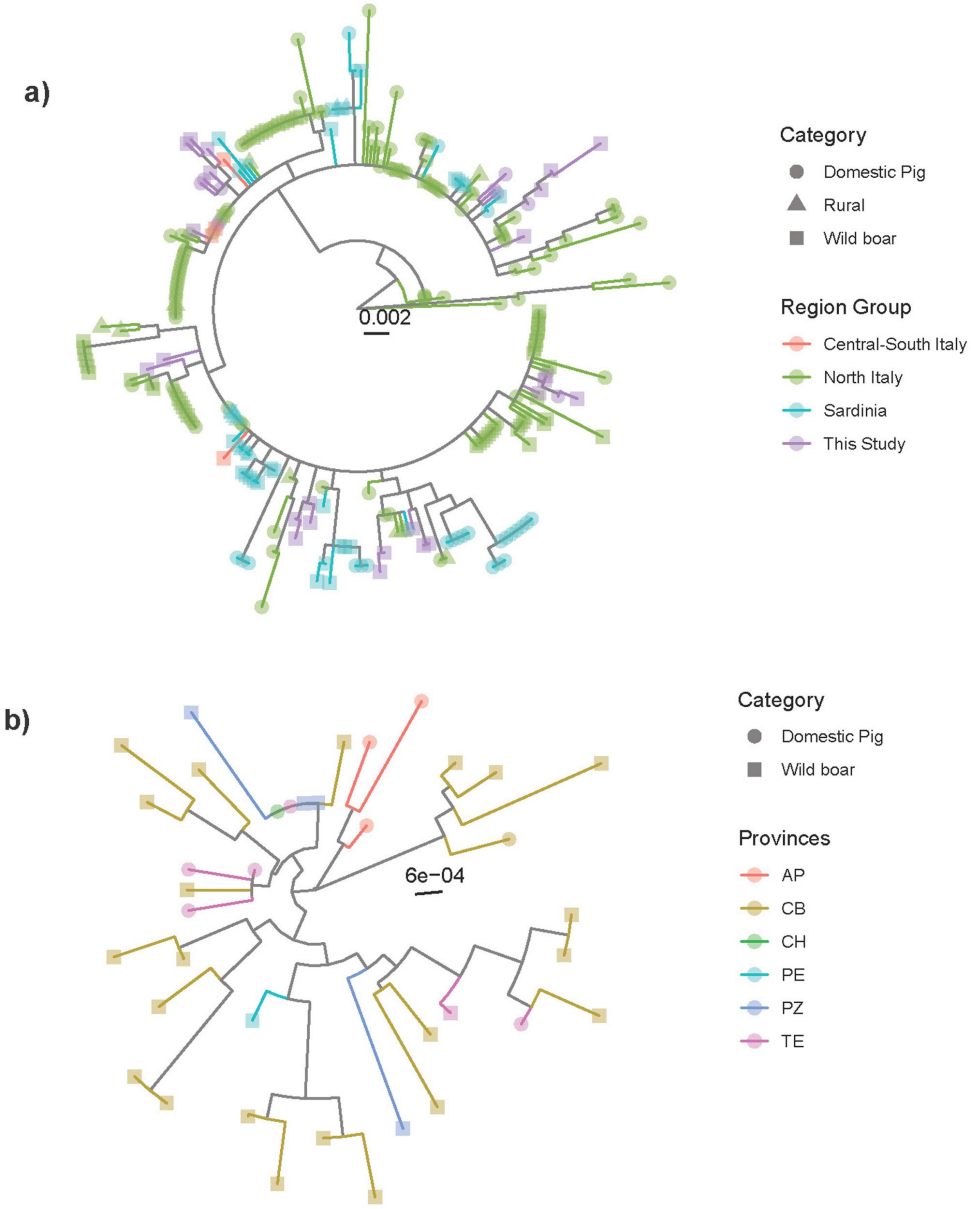
(**a**) Phylogenetic tree based on the ORF2 of the Italian PCV2d strain. Tips have been coloured according to the region of origin while the host population category has been depicted by different signs. (**b**) Phylogenetic tree based on the ORF2 of the PCV2d strain obtained from Central-Southern Italina provinces. Tips have been coloured according to the province of origin while the host population category has been depicted by different signs. AP = Ascoli Piceno, CH = Chieti, CB = Campobasso, PE = Pescara, PZ = Potenza, TE = Teramo

A total of 36 complete genome sequences were obtained mapping the consensus sequence with reference strain HN-BF-2016 (GenBank accession number MK604479.1). Strains originated from 10 pigs and 38 wild boars located in 3 regions and 5 provinces between 2020 and 2023. The median number of raw reads obtained was 4,501,328 (range: 1,519,188- 8,096,060), while the median number of mapped reads was 104,926.3 (range: 52,215–279,410). The obtained sequences are available in GenBank (accession numbers PQ468838–PQ468873) (Supplementary Table 2). All sequences were classified in the PCV2d genotype, with the exception of strains PQ468869 (PCV2a) and PQ468849 (PCV2b) (Supplementary Fig. 1). No evidence of within-dataset recombination was detected. Comparison of these strains with a dataset of international sequences showed the closest relationship with sequences from Asia and Europe for PQ468869 and with another Italian strain for PQ468849. Phylogenetic analysis of PCV2d sequences and comparison with previously sequenced Italian strains demonstrated that the strains sequenced in the present study—and more broadly, strains from Central-Southern Italy—were interspersed throughout the phylogenetic tree, forming small clades or even independent branches. These groups typically originated from clusters predominantly containing sequences from Northern Italian commercial farms, although sequences from rural farms or wild boars were also present. Although more sequences were obtained from wild boars, in most Central-Southern Italy clusters, at least one sequence originating from a domestic pig was present in most instances (Fig. 2 and Supplementary Fig. 2). The analysis focusing on strains sampled Central Italy revealed some geographical clustering, although closely related sequences occasionally originated from different provinces among both domestic pigs and wild boars (Fig. 2b).

### PCV3 full genome sequencing and phylogenetic analysis

**Fig. 3:**
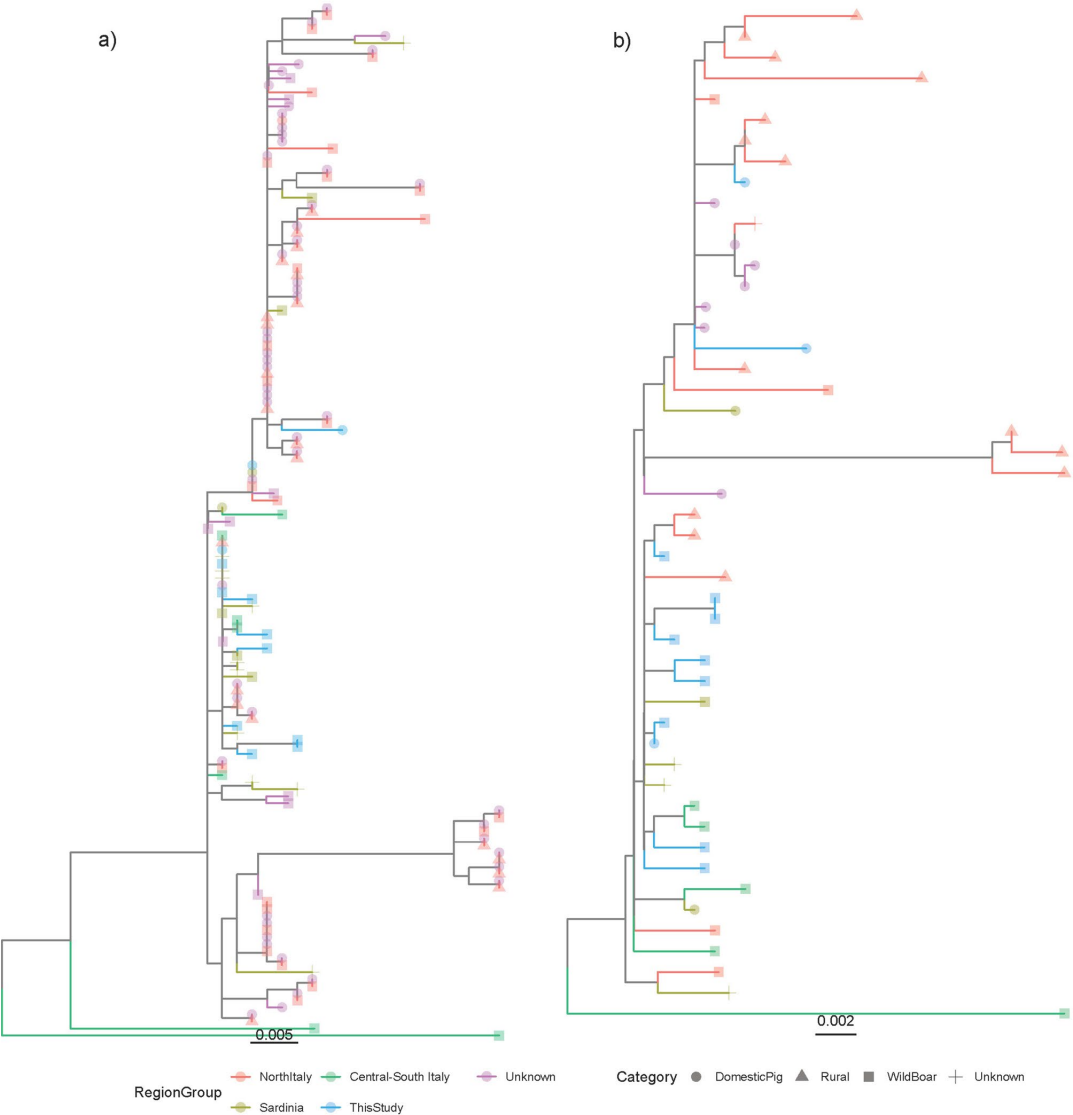
Phylogenetic tree based on the ORF2 (**a**) and complete genome (**b**) of the Italian PCV3 strain. Tips have been coloured according to the region of origin while the host population category has been depicted by different signs

Twelve complete genome sequences were obtained (Supplementary Table 2), mapping the consensus sequence with reference strain PCV3SAR2 (GenBank accession number MN781188.1). The median number of raw reads obtained was 3,334,967.88 (range: 1,050,754- 6,946,720), while the median number of mapped reads was 180,842.53 (range: 11,770 − 433,181). The sequences, all belonging to PCV3a genotype, originate from 9 wild boars and 3 domestic pigs, collected from 3 regions and 3 provinces between 2020 and 2023. The phylogenetic analysis based on the ORF2 dataset showed that sequences obtained in the present study are dispersed throughout the phylogenetic tree, forming distinct clades. Although most sequences were obtained from wild boars, in a few cases, they were identical to sequences sampled from domestic pigs in Central-Southern Italy, which, in turn, were identical to sequences from intensively and rurally raised pigs sampled in Northern Italy. However, other wild boar strains formed clusters exclusively composed of wild boar sequences or had the closest relationship with sequences previously sampled from wild boars in Central-Southern Italy or Sardinia (Fig. 3 and Supplementary Fig. 3). These findings were largely confirmed by the complete genome analysis, although the lower number of available sequences reduced the resolution of the analysis (Fig. 3 and Supplementary Fig. 4).

## Discussion

The presence and circulation of PCV2 in Italy have been documented and updated over time, revealing multiple genotypes, including PCV2a, PCV2b, PCV2d, and PCV2e, with a clear trend towards the progressive replacement of PCV2a and PCV2b by PCV2d in both domestic and wild boar populations [9, 30, 31]. Interestingly, a distinctive PCV2e population appears almost exclusively established in rurally raised animals [32]. In recent years, the epidemiological scenarios for PCV3 have also been explored, showing wide circulation in both domestic and wild boar populations. Besides the role of wild boars, backyard or non-integrated pig farms have been shown to play a significant role in the epidemiology of PCVs, albeit with some differences between PCV2 and PCV3 [12]. Nevertheless, much of this knowledge has originated from studies conducted in Northern Italy. Regions in Central-Southern Italy, however, have been largely neglected.

Overall, the present study describes a scenario largely overlapping with those previously observed in Northern Italy. A high detection frequency, around 40% for both PCV2 and PCV3, was observed in both host populations, with an even higher prevalence in wild boars, supporting the relevance of these populations in PCVs epidemiology. Unfortunately, only a subset of the strains identified by PCR could be successfully sequenced. This limitation is commonly observed and is primarily due to the lower sensitivity of sequencing methods compared to PCR-based diagnostics. It is important to consider that this discrepancy may introduce a bias, potentially favoring the sequencing of strains with higher replication efficiency, which could in turn be associated with increased pathogenicity. This aspect warrants careful consideration and highlights the need for improved sequencing strategies in future studies.

In Central-Southern Italy also, a predominance of PCV2d was observed. Although a lack of historical data prevents us from evaluating trends over time, a genotype shift in these regions, likely mirroring or following that in Northern Italy, can be hypothesized [9, 30]. The presence of multiple clades interspersed within the phylogenetic tree confirms extensive and unrestrained viral circulation across Italian regions, with the same region harbouring multiple strains and identical strains found in distant areas. Given that most Central-Southern Italy clades stem independently from other groups containing sequences from intensively raised pigs in Northern Italy, multiple introduction events are likely, also involving wild boars. While several clusters primarily included wild boar sequences (likely due to the higher number of strains obtained from this host), most clades comprised at least one sequence from domestic pigs. Based on this evidence and the limited host range of wild boars, long-range viral spread mediated by commercial pigs, followed by “escape” into the wild, seems more plausible than the reverse [9].

Subsequently, PCV2 was proven able to circulate persistently in wild populations, as shown by the prolonged detection of similar, yet not identical, strains in wild populations. While closely related sequences generally originated from the same province, exceptions were noted, suggesting some mobility, migration, or range overlap among host populations. Accordingly, comparable detection frequency featured different regions, supporting panmictic population, as well as comparable farming and environmental conditions. Interestingly, similar findings were observed for PCV3, where sequences obtained in this study often clustered with other strains previously sequenced from wild boars in Central-Southern Italy [15]. However, when Central-Southern Italy sequences were analysed in greater detail, no strong geographical clustering was detected, indicating significant local strain migration. Thus, the apparent linkage among wild populations may be mediated by domestic farms that were not detected due to limited sample availability.

Establishing a link with strains from Sardinia is more complex. Previous research has suggested strain migration from the mainland to Sardinia, potentially facilitated by the importation of runts from intensive farms in Northern Italy to rural Sardinian farms, which are commonly used for traditional dishes [30]. Therefore, the observed similarity between Sardinian and Central-Southern Italy strains may be due to a shared origin of pig—and thus virus—introduction. Although a comprehensive analysis of the international context was beyond the scope of this study, a preliminary evaluation suggests Italian strains, including those from Southern Italy, are part of the international mixing of PCV2 strains [7]. Considering the higher industrialization and connection network of the pig industry in Northern Italy, new strains are more likely introduced in this region, with subsequent southward spread, though alternative scenarios cannot be excluded. The epidemiological scenario for PCV3 was broadly similar, indicating shared epidemiological forces and determinants. However, the smaller number of available sequences and the higher sampling bias toward wild boars reduce the reliability of inferences regarding viral distribution and dispersal patterns. Nonetheless, the presence of identical or closely related strains in northern and southern Italian domestic pigs, and among southern domestic and wild boars, suggests a pattern similar to that observed for PCV2.

## Conclusions

The findings of this study further confirm the broad presence, circulation, and genetic variability of both PCV2 and PCV3 in Italy, including in Central-Southern Italian regions, where pig production is less developed. Both wild animals and rural or non-integrated production systems are deeply connected to the industrialized North on a large geographical scale, although precise links and dispersal patterns are challenging to establish. While assessing the local economic impact of PCVs was beyond the scope of this work, given the relevance of these pathogens, continuous monitoring should be supported to prevent their detrimental effects on a developing sector. Furthermore, the extensive circulation of these pathogens in wild boars and their connections with domestic pigs represent a potential threat due to the often lower external biosecurity standards of non-integrated production systems. This situation is particularly concerning for the persistence, spread, and evolution of the virus, and because it serves as a sentinel for effective epidemiological contacts between wild and domestic populations. Such contacts could have far more severe consequences for more challenging infections, such as African swine fever, recently introduced and spread in Northern Italy.

**Table 1:** Summary table reporting number of positive animals categorized according to the considered pathogen, host species and collection region

Region	PCV2			PCV3^a^		
	Pig	Wild boar	Total	Pig	Wild boar	Total
Abruzzo	28/84 (33.33%)	5/11 (45.45%)	33/95 (34.74%)	5/20 (25%)	1/7 (16.67%)	6/27 (11.11%)
Molise	13/46 (28.26%)	67/105 (63.81%)	80/151 (52.98%)	3/10 (33.33%)	27/55 (49.09%)	30/65 (46.15%)
Marche	8/13 (61.54%)	-	8/13 (61.54%)	2/8 (25%)	-	2/8 (25%)
Total	50/143 (34.96%)	72/116 (62.07%)	122/259 (47.10%)	10/38 (26.31%)	28/62(45.16%)	38/100 (38%)

^a^ Only samples collected between 2023 and 2024 and those collected before 2023 that tested positive for PCV2 were tested for PCV3

**Table 2:** Summary table reporting number of positive samples to individual pathogens or co-infection, categorized according to the host species

Animal species	PCV2	PCV3^a^	PCV2 + PCV3
Pigs	50/143 (34.97%)	10/38 (26.32%)	6/38 (15.79%)
Wild boars	72/116 (62.07%)	28/62 (46.16%)	23/62 (37.1%)
Total	122/259 (47.1%)	38/100 (38%)	29/100 (29%)

^a^ Only samples collected between 2023 and 2024 and those collected before 2023 that tested positive for PCV2 were tested for PCV3

## Electronic supplementary material

Below is the link to the electronic supplementary material.

**Supplementary Material 1**: **Supplementary Fig. 1.** Phylogenetic tree based on the ORF2 of the strain obtained in the present study plus a set of reference strains obtained from Franzo et al., 2018 [6]. Strain metadata, including collection host, region, province and date have been provided in the sequence name. The bootstrap support (> 70) is reported nearby the corresponding node.

**Supplementary Material 2**: **Supplementary Fig. 2.** Phylogenetic tree based on the ORF2 of the Italian PCV2 strain. Strain metadata, including collection host, region and date have been provide in the sequence name. Tips have been coloured according to the host population category. Sequences obtained in the present study have been marked with a full circle. The bootstrap support (> 70) is reported nearby the corresponding node.

**Supplementary Material 3**: **Supplementary Fig. 3.** Phylogenetic tree based on the ORF2 of the Italian PCV3 strain. Strain metadata, including collection host, region and date have been provide in the sequence name. Tips have been coloured according to the host population category. Sequences obtained in the present study have been marked with a full circle. The bootstrap support (> 70) is reported nearby the corresponding node.

**Supplementary Material 4**: **Supplementary Fig. 4.** Phylogenetic tree based on the complete genome of the Italian PCV3 strain. Strain metadata, including collection host, region and date have been provide in the sequence name. Tips have been coloured according to the host population category. Sequences obtained in the present study have been marked with a full circle. The bootstrap support (> 70) is reported nearby the corresponding node.

**Supplementary Material 5**: **Supplementary table 1**

**Supplementary Material 6: Supplementary table 2**

## Data Availability

The datasets generated and analysed during the current study are available in the Genbank ( PQ468838- PQ468885).
