# Supplementary material for: Molecular epidemiology and genetic characterization of PCV2 and PCV3 circulating in domestic pigs and wild boars in central-southern regions of Italy
Source: BMC Vet Res. 2025 Jul 21;21:478. doi: 10.1186/s12917-025-04928-0 (PMC12278492; doi:10.1186/s12917-025-04928-0)
Supplement: Supplementary file 5 — Supplementary Material 5: Supplementary table 1 [file 12917_2025_4928_MOESM5_ESM.docx]

| Host | Pathogen | 2020 | 2021 | 2022 | 2023 | 2024 | Total |
| --- | --- | --- | --- | --- | --- | --- | --- |
| Domestic pig | PCV2 | 29 | 45 | 33 | 30 | 6 | 143 |
|  | PCV3 | 3 | 6 | 2 | 21 | 6 | 38 |
| Wild boar | PCV2 |  | 1 | 42 | 68 | 5 | 116 |
|  | PCV3 |  | 1 | 12 | 44 | 5 | 62 |

Supplementary table 1. Table showing Domestic pigs and wild boar samples tested for each pathogen per year
