# Supplementary material for: Molecular epidemiology and genetic characterization of PCV2 and PCV3 circulating in domestic pigs and wild boars in central-southern regions of Italy
Source: BMC Vet Res. 2025 Jul 21;21:478. doi: 10.1186/s12917-025-04928-0 (PMC12278492; doi:10.1186/s12917-025-04928-0)
Supplement: Supplementary file 2 — Supplementary Material 2: Supplementary Fig. 2. Phylogenetic tree based on the ORF2 of the Italian PCV2 strain. Strain metadata, including collection host, region and date have been provide in the sequence name. Tips have been coloured according to the host population category. Sequences obtained in the present study have been marked with a full circle. The bootstrap support (> 70) is reported nearby the corresponding node. [file 12917_2025_4928_MOESM2_ESM.pdf]

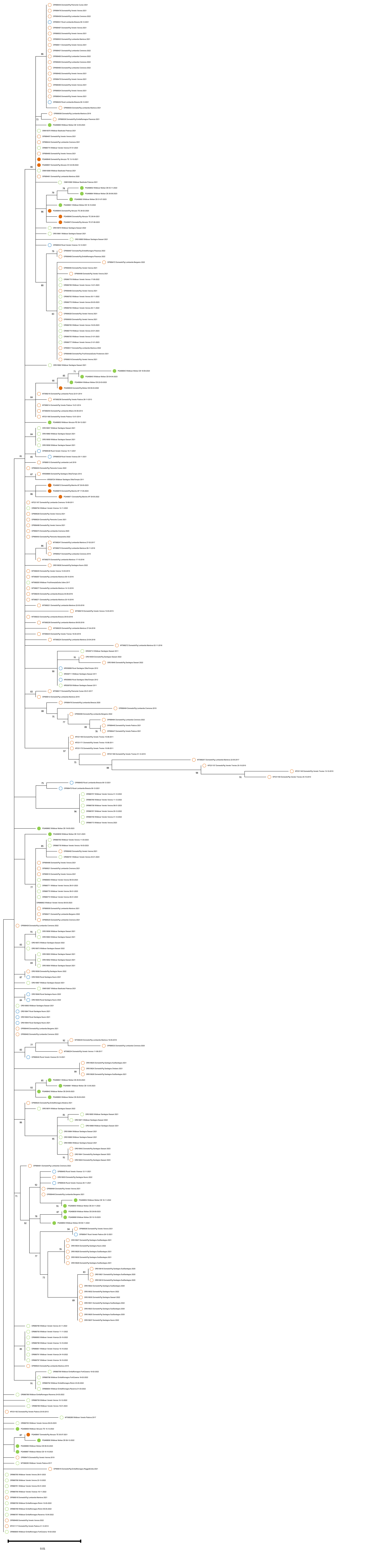
0.02
